# Exploring the Role of Axons in ALS from Multiple Perspectives

**DOI:** 10.3390/cells13242076

**Published:** 2024-12-17

**Authors:** Xiaosu Chen, Shuchang Lv, Jinmeng Liu, Yingjun Guan, Chunjie Xu, Xiaonan Ma, Mu Li, Xue Bai, Kexin Liu, Haoyun Zhang, Qiupeng Yan, Fenghua Zhou, Yanchun Chen

**Affiliations:** 1 Department of Histology and Embryology, Shandong Second Medical University, Weifang 261053, China; chenxiaosu85@163.com (X.C.); 19863823029@163.com (S.L.); xu1261781793@163.com (C.X.); 18855241172@163.com (X.M.); bx13176966202@163.com (X.B.); 2 Neurologic Disorders and Regenerative Repair Lab of Shandong Higher Education, Shandong Second Medical University, Weifang 261053, China; 18353687185@163.com (J.L.); limu1009@gmail.com (M.L.); 18706618772@163.com (K.L.); haoyunzh@sdsmu.edu.cn (H.Z.); yanqiupeng@sdsmu.edu.cn (Q.Y.); zhoufh@sdsmu.edu.cn (F.Z.); 3 Department of Physiology and Pathophysiology, School of Basic Medicine, Qingdao University, Qingdao 266000, China

**Keywords:** amyotrophic lateral sclerosis, axon degeneration, axon transport, axon regeneration, therapy

## Abstract

Amyotrophic lateral sclerosis (ALS), commonly known as motor neuron disease, is a neurodegenerative disorder characterized by the progressive degeneration of both upper and lower motor neurons. This pathological process results in muscle weakness and can culminate in paralysis. To date, the precise etiology of ALS remains unclear. However, a burgeoning body of research indicates that axonal dysfunction is a pivotal element in the pathogenesis of ALS and significantly influences the progression of disease. Dysfunction of axons in ALS can result in impediments to nerve impulse transmission, leading to motor impairment, muscle atrophy, and other associated complications that severely compromise patients’ quality of life and survival prognosis. In this review, we concentrate on several key areas: the ultrastructure of axons, the mechanisms of axonal degeneration in ALS, the impact of impaired axonal transport on disease progression in ALS, and the potential for axonal regeneration within the central nervous system (CNS). Our objective is to achieve a more holistic and profound understanding of the multifaceted role that axons play in ALS, thereby offering a more intricate and refined perspective on targeted axonal therapeutic interventions.

## 1. Introduction

Amyotrophic lateral sclerosis (ALS) is a fatal neurodegenerative disease that affects motor neurons. It is characterized by the progressive degeneration of both upper and lower motor neurons, primarily affecting the spinal cord and brain stem. This degeneration ultimately results in progressive skeletal muscle weakness, respiratory muscle paralysis, and respiratory failure [1,2]. Research indicates that, in addition to motor neurons, other types of neurons, such as sensory neurons [3,4,5] and interneurons [6,7], are also affected in the progression of ALS disease. However, this review focuses more on the role of motor neurons in the development of ALS.

Typically, the average age of onset for ALS is between 51 and 66 years [8], with a post-diagnosis life expectancy of only 2–5 years [9]. ALS is broadly classified into familial and sporadic subtypes, of which the most common ALS mutant genes are the copper-zinc superoxide dismutase (*SOD1*), chromosome 9 open reading frame 72 (*C9ORF72*), fused in sarcoma (*FUS*), and TAR DNA-binding protein (*TARDBP*) [10]. The cumulative effect of these genetic mutations is notably significant, accounting for a considerable fraction of ALS incidences, with approximately 48% of familial ALS cases and 5% of sporadic ALS cases being attributable to these specific genetic changes [11,12].

The understanding of the pathogenesis of ALS primarily encompasses oxidative stress; dysregulation of mitochondria, protein homeostasis, RNA processing, axonal transport, and nuclear-cytoplasmic transport (NCT); neuroinflammatory responses; excitotoxicity; and DNA lesions [13]. Notably, ALS progression is not likely the result of a single causative factor, indicating a multifaceted interplay among these mechanisms in the disease’s development. Over the past few decades, the prospect of diagnosis and treatment in ALS has been exceedingly grim. To date, only four medications—riluzole [14], edaravone [15], relyvrio [16], and tofersen (Qalsody) [17]—have been approved by the FDA. Although these drugs can alleviate some symptoms to a certain extent, they are incapable of curing the disease or halting its progression. Consequently, the urgency to discover an effective treatment is paramount.

Axons, the extended cytoplasmic projections of neurons, serve as essential conduits for the transmission of nerve impulses to other neurons or to effectors, including muscles and glands [18]. They play a crucial role in ensuring the orderly and directional transport of signals over long distances within the neurons. In ALS, axonal injury is a pivotal pathological feature, manifesting as impaired axonal regrowth and transport functions, closely linked to the disease’s progression. A comprehensive understanding of the role of axons in ALS is crucial for devising targeted treatment strategies.

This paper aims to provide a comprehensive review of axonal ultrastructure and elucidate the underlying molecular mechanisms of axon degeneration. Additionally, we will examine the challenges posed by axon transport barriers and explore the possibilities for axon regeneration within the CNS. The overarching aim is to enhance our understanding of the critical role axons play in ALS and to establish a solid foundation for the formulation of innovative therapeutic approaches.

## 2. The Ultrastructure of the Axon

The axon is a slender and elongated neuronal process primarily responsible for the transmission of information and the transport of materials between neurons or between neurons and their surrounding effector organs, and the maintenance of these functions is contingent upon the axon’s stable internal architecture (Table 1). Microfilaments (MFs), microtubules (MTs), and intermediate filaments (IFs) interlace to form the cytoskeleton, which maintains cellular structure and function [19].

MTs, which consist of numerous α- and β-tubulin heterodimers, exhibit significant polarity. The “plus” end grows more rapidly, while the “minus” end grows more slowly. These structures undergo continuous polymerization and depolymerization at the centrosome, thereby enhancing axonal rigidity and providing a scaffold for intracellular transport [20]. In essence, MTs serve a role akin to “rails” within the axon, facilitating the movement of organelles and vesicles.

Neurofilaments (NFs), the primary cytoskeletal proteins of the axon, belong to the family of IFs. They are composed of three distinct subunit proteins: the neurofilament light (NF-L), medium (NF-M), heavy (NF-H) proteins. These subunits assemble into rod-shaped structures with a diameter of 10 nanometers, equipped with radial side arms, creating a structure reminiscent of a bottle brush. The attractive forces between the NF-M side arms result in the formation of a compact nematic gel, whereas the repulsive forces of the NF-H side arms facilitate the formation of a looser isotropic gel. This suggests that NFs may exhibit self-organizing properties akin to those of liquid crystals [21]. Research has demonstrated that NFs are instrumental in both promoting the radial expansion of axons [22] and maintaining axon diameter [23].

**Table 1:** The ultrastructure of the axon.

Ultrastructure	Composition	Characteristics	Main Function	Refs.
MTs	α-; β-tubulin heterodimers	Significant polarity	Enhance axonal rigidity and provide a scaffold for intracellular transport	[20]
NFs	NF-L; NF-M; NF-H	Self-organizing properties akin to those of liquid crystals	Promote the radial expansion of axons and maintain axon diameter	[22,23]
MFs	Actin	Double-helical fibers with a diameter of approximately 7 nanometers	Provide internal mechanical support, serve as tracks for the intracellular trafficking of materials, and exert forces to facilitate cell motility	[24]
Other components of axoplasm	Mitochondria, fragments of the endoplasmic reticulum, Golgi apparatus, and various other organelles and molecules necessary for axonal function	Physical properties are similar to those of structured gels or liquid crystals	Assist in maintaining the shape and integrity of axons	[25]

MFs are double-helical fibers polymerized by actin, with a diameter of approximately 7 nanometers, and they are pivotal in providing internal mechanical support, serving as tracks for the intracellular trafficking of materials, and exerting forces to facilitate cell motility [24].

Axoplasm refers to the internal cytoplasm of axons, which, in addition to the cytoskeletal components, also includes mitochondria, fragments of the endoplasmic reticulum, Golgi apparatus, and various other organelles and molecules necessary for axonal function. The physical properties of axoplasm are similar to those of structured gels or liquid crystals, which assist in maintaining the shape and integrity of axons [25].

## 3. Axonal Damage, Closely Related to the Pathology of ALS

### 3.1. Pathological Manifestations of Axonal Damage

Abnormal elevations of NF proteins in extracellular fluids, including cerebrospinal fluid (CSF) and peripheral blood, serve as biomarkers for axonal damage across various pathological conditions, such as neurodegenerative, inflammatory, and traumatic diseases that disrupt neurofilament homeostasis [26]. In the context of ALS, research has identified significantly increased levels of NF-L [27] and NF-H [28] in the CSF of patients. Intriguingly, NF-H levels are notably elevated in patients with rapidly advancing ALS compared to those with a slower progression, hinting that NF-H may mirror the severity of axonal injury to a certain degree.

Paralleling these clinical observations, the hSOD1-G93A ALS mouse model exhibits axonal damage, myelin degeneration, and macrophage infiltration in both the ventral root (VR) and dorsal root (DR) [29]. Light and electron microscopic examinations have further uncovered the presence of degraded and swollen spinal axons at early and pre-symptomatic stages in SOD1 (H46R) ALS mice, with an accompanying buildup of granular aggregates and autophagosome-like vesicles [30]. These findings underscore the early onset of axonal pathology in ALS. Moreover, high-resolution diffusion tensor imaging (DTI) has successfully pinpointed early signs of spinal white matter axonal pathology in an ALS mouse model, offering valuable insights into the neurodegenerative processes at the core of the disease [31].

### 3.2. Risk Factors of Axonal Damage 

**Figure 1:**
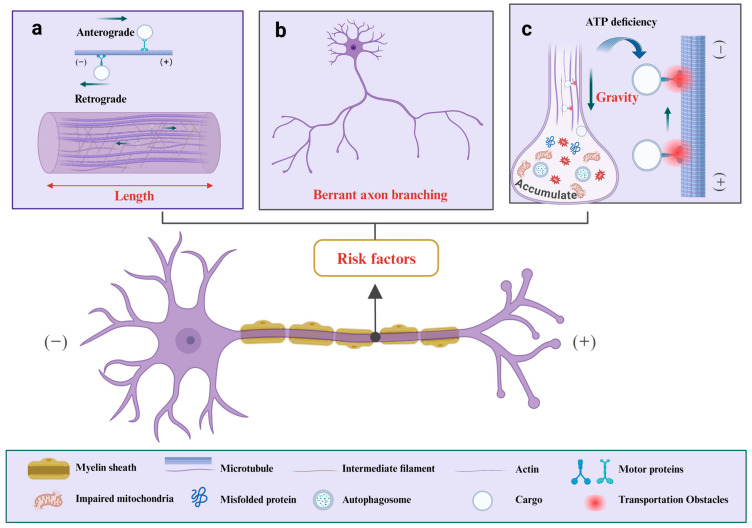
Axonal damage can be influenced by a variety of risk factors. (**a**) The length of axon could be a contributing factor to the potential for damage. (**b**) Abnormal axonal branching is implicated in ALS pathogenesis. (**c**) Under the influence of gravity, the ATP required by most longitudinal axons to reverse transport metabolic waste back to the cell body is greater than that required by lateral axon transport. This disparity can result in a progressive accumulation of waste at the axonal termini, which, if not promptly recycled, may lead to compromised axonal transport function. The red bidirectional arrow delineates the range; the black arrow highlights the risk factors; the dark green arrows signify direction, and the blue arrows with arcs point to a magnified view of axonal transport impediments resulting from ATP deficiency.

As the conduits for neuronal output, axons can extend over a meter in length, indicating that damage to any segment can impair both the structure and function of the axon, a factor intimately linked to the progression of ALS [32] (Figure 1a). This concept aligns with Tallon et al.’s proposition that axonal degeneration in motor neurons, particularly in type II fibers of SOD1 mice, is an axon-length-dependent process [33].

Healthy upper motor neurons (UMNs), characterized by longer axons and enhanced branching or dendritic arborization, can be assessed for their potential to improve neuronal health across a spectrum of neuronal types. An analogue of cyclohexane-1,3-dione, NU-9, has been shown to elongate axons and promote enhanced branching and dendrification, especially when co-administered with riluzole or edaravone [34]. However, abnormal increase in axon branching is implicated in ALS pathogenesis (Figure 1b), as the expression of mutant SOD1^G93A^ in adult motor neurons can enhance axonal growth and branching [35]. Fos-B, a transcription factor belonging to the activation protein-1 (AP-1) family, plays a crucial role in the development and functionality of the nervous system. Studies indicate that motor neurons with FUS mutations exhibit aberrant increase in axonal branching [36], which can be mitigated through modulating Fos-B levels [37]. 

In addition, Akaishi hypothesizes that under gravitational influence, the ATP required for the retrograde transport of metabolic waste in longitudinal axons would exceed that needed for transverse transport, potentially leading to the accumulation of unrecycled waste at the axon terminals and contributing to the loss of motor neurons [38] (Figure 1c).

## 4. Exploring Axonal Degeneration Pathways and the Underlying Molecular Mechanisms for Axonal Protection in ALS

Nerve injuries resulting from ligation, transection, or other causes often lead to a characteristic sequence of events. Distal to the site of injury, nerve fibers progressively enlarge and then exhibit a beaded and fragmented appearance. Ultimately, these damaged structures are phagocytosed and cleared by surrounding glial cells and macrophages. 

The process now recognized as Wallerian degeneration was initially described by Augustus Volney Waller in 1850 [39]. As our understanding of degenerative mechanisms has advanced, it has become evident that axonal degeneration in neurodegenerative diseases, a process known as “Wallerian-like” degeneration, may exhibit characteristics similar to Wallerian degeneration [40,41].

Sterile alpha and Toll/interleukin-1 receptor motion-containing protein 1 (SARM1) is a core executor of pathologic axon degeneration, with its NADase activity playing an essential role in promoting Wallerian axonal degeneration [42]. The SARM1 protein is composed of several distinct domains: the Armadillo/HEAT motif (ARM) domain, the sterile alpha motif (SAM) domain, and the Toll/interleukin-1 receptor (TIR) domain. The TIR domain exerts a neurodegenerative effect through NADase activity, while the SAM domain contributes to the formation of octamers [43]. The ARM domain interacts specifically with the αA helix and BB ring of the TIR domain through particular amino acid residues, thereby modulating NADase activity [44]. Two distinct structural conformations of SARM1 have been delineated by cryo-electron microscopy (cryo-EM) technique: the self-inhibited and active states [45]. In the self-inhibited state, SARM1 forms a compact octameric structure. Mutation analysis of five different interfaces shows that these interfaces are necessary for self-inhibition, and point mutations at each interface will lead to constant activation of SARM1. Notably, in healthy neurons, SARM1 is consistently maintained in a self-inhibited state. However, in ALS patients, specific allelic variants of SARM1 are observed, which encode for SARM1 proteins that harbor mutations within the ARM domain [46,47]. These mutations trigger an abnormal increase in SARM1 NADase activity, eventually leading to axonal loss [47]. 

**Figure 2:**
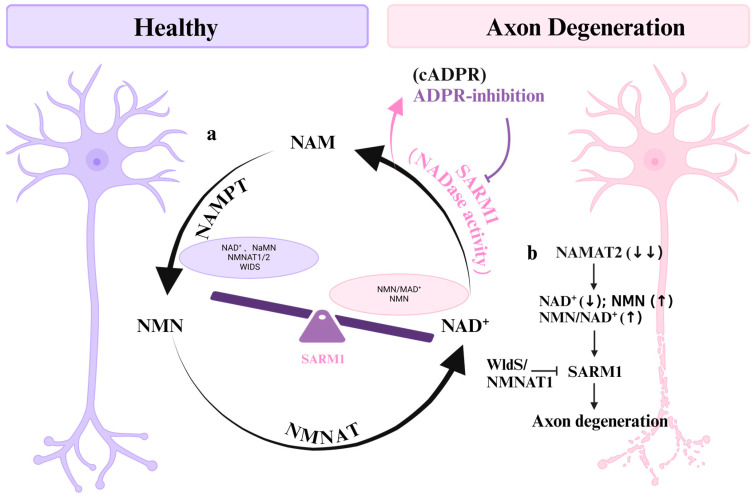
The activity of SARM1 is intricately regulated by NAD^+^ metabolism. (**a**) NMN and NAD^+^ are known to respectively activate and inhibit the NADase activity of recombinant SARM1. NMN facilitates the degradation of remaining NAD^+^ through the activation of SARM1’s NADase activity, resulting in the production of NAM and ADPR (cADPR), which ultimately triggers the degeneration of axons. (**b**) The deletion of NMNAT2 triggers the activation of SARM1 through the modulation of NAD^+^, NMN, and the NAD^+^/NMN ratio, leading to the degeneration of axons. This degenerative process can be counteracted by the elevated expression of WldS/NMNAT1. The black and pink arrows indicate direction, the purple arrows denote suppression, and the black arrows within parentheses signify trends.

The local metabolic environment plays a pivotal role in regulating the activity of SARM1. In particular, Nicotinamide Mononucleotide (NMN) and Nicotinamide Adenine Dinucleotide (NAD^+^) have been shown to competitively bind to the same allosteric site within the ARM domain of recombinant SARM1, thereby activating and inhibiting its NADase activity, respectively [44,45,48,49]. Studies have shown that these environmental triggers of SARM1, such as NMN, may interact with other ALS risk factors, ultimately contributing to the development of ALS [50]. By binding to this allosteric pocket in the N-terminal autoregulatory ARM domain, NMN triggers conformational shifts that activate the NADase activity located in the C-terminal TIR domain. This activation process results in the degradation of residual NAD^+^ into Nicotinamide (NAM) and ADP-ribose (ADPR), ultimately leading to axonal degeneration [51] (Figure 2a).

Conversely, inhibiting Nicotinamide phosphoribosyltransferase (NAMPT) can safeguard damaged axons and synapses by preventing the buildup of NMN [52]. Bratkowski and colleagues suggested that the NAD-dependent active site inhibitor of SARM1 functions by interrupting the hydrolysis of NAD and covalently linking it to the reaction product ADPR [53]. Notably, cyclic ADP-ribose (cADPR) serves as a direct biomarker of SARM1 activity, working in tandem with NF-L, which indicates distal damage due to SARM1-dependent axon breakage [54]. Employing this dual biomarker strategy will significantly enhance research into SARM1 activity in neurodegenerative diseases and validate targets for SARM1 inhibitors in animal models and human studies.

Studies have shown that SARM1 is activated when the ratio of NMN to NAD^+^ increases, thereby inducing axonal degeneration [55,56]. Furthermore, axonal degeneration can be effectively mitigated by modulating NAD^+^-related metabolites. For instance, nicotinic acid mononucleotide (NaMN), which is the deaminated derivative of NMN, has the ability to compete with NMN for binding to the allosteric site of SARM1, thus blocking its activation [57]. NAD^+^ biosynthesis involves a crucial enzyme, nicotinamide mononucleotide adenylyltransferase (NMNAT), which plays a central part in sustaining neuronal health. Significantly diminished levels of both NMNAT1 and NMNAT2 can trigger the activation of SARM1, consequently initiating a cascade of events that lead to axonal degeneration [58,59]. Notably, NMNAT1 does not alter the synthesis of NAD^+^; rather, it prevents the SARM1-dependent consumption of NAD^+^ induced by damage [60,61]. The axonal degeneration resulting from the depletion of NMNAT2 can be counteracted by the overexpression of WldS/NMNAT1, thereby demonstrating a neuroprotective effect [62,63,64,65] (Figure 2b).

Wnk kinase is a novel signaling component that provides protective functions in both developmental and adult axons. The Drosophila Wnk kinase (dWnk), along with its mammalian orthologs WNK1/2, synergistically interacts with NMNAT to counteract the destabilizing effects of axonal factors D-Sarm and Axed [66,67]. Consequently, overexpression of dWnk and WNK1/2 can prevent the axonal degeneration induced by D-Sarm and Axed.

Previous research has made substantial progress in elucidating the molecular mechanisms underlying Wallerian degeneration, which is considered a pivotal feature in a spectrum of neurodegenerative diseases. Small molecule inhibitors that target SARM1, such as the isothiazole class of compounds, present a promising therapeutic approach for preventing axonal degeneration and facilitating the regeneration of damaged axons. This could have a profound impact on the management of axonal disorders within both the CNS and peripheral nervous system (PNS) [68,69,70].

## 5. Axonal Transport Disorders Constitute an Important Part of the Pathogenesis of ALS

### 5.1. Normal Axonal Transport

Axons are primarily responsible for the transmission of nerve impulses from the soma of neurons to their respective postsynaptic elements, which include other neurons, muscle cells, or glandular cells. Moreover, axonal transport can move cargo either away from or towards the cell body or the proximal end of the axon. This directional movement is typically categorized into anterograde and retrograde transport activities [71]. Anterograde transport facilitates the movement of essential components, such as synaptic vesicle precursors, mitochondria, signaling endosomes, autophagosomes, lysosomes, and mRNA particles, as well as the clearance of cellular metabolic waste [72,73]. The movement is commonly facilitated by kinesin family member 5A (KIF5A), alternatively referred to as kinesin-1, which belongs to the motor protein family [74,75]. In contrast, retrograde transport relies on the adaptor complex dynactin to recognize and link specific organelles and cargo to dynein. Dynein then propels the transport of nutrients, growth factors, damaged organelles, signaling molecules, and secretions back towards the cell body, also playing a role in the removal of misfolded and aggregated proteins [75,76]. Notably, another adaptor protein, the mitochondrial Rho GTPase 1 (Miro), can link the surface of mitochondria to the microtubule motor protein Milton [77]. Disruption of this interaction can selectively impair the efficacy of both anterograde and retrograde transport mechanisms mediated by motor proteins [78]. The precise delivery of specific cargo to designated axonal locations is contingent upon an anchoring protein structure, which serves as a critical component of the transport system. For instance, syntaphilin (SNPH) engages in SNPH-KIF5 coupling through the Miro-Ca^2+^ sensor, anchoring mitochondria to the microtubule “track” and ensuring accurate mitochondrial transport [79].

### 5.2. Impaired Axonal Transport of Mitochondria

Mitochondria are the most studied cargo along the axon, and most of the current knowledge about axonal transport comes from this organelle. As the center of cellular energy production, the structure of mitochondria primarily consists of two functionally distinct membranes: the outer mitochondrial membrane (OMM) and the inner mitochondrial membrane (IMM) [80]. The IMM is functionally crucial as it contains the respiratory chain and FoF1 ATP synthase, playing a significant role in the process of oxidative phosphorylation [81]. Within axons, the majority of mitochondria are stationary, with only a minority (30–40%) being mobile [78]. However, under special circumstances, mitochondria become highly concentrated in areas with high energy demands, such as growth cones [82] and axon bifurcation [83], to maintain energy supply. Axonal transport is inherently energy-intensive, relying on mitochondria, the cell’s renowned “powerhouses”, to provide the necessary energy. Impairment or damage to these organelles can disrupt the energy supply, consequently resulting in deficits of axonal transport. For example, studies in *C9ORF72*-ALS mice have found that loss of mitochondrial function leads to axonal dysfunction, which is mainly reflected in two phenotypes: shorter axon length and impaired rapid axonal transport of mitochondrial cargo [84]. Anterograde axonal transport delivers mitochondria to their anchored positions, while retrograde transport is responsible for the removal of damaged mitochondria. When mitochondria are damaged, the PINK1/PARKIN signaling pathway is activated, and Parkin marks the damaged mitochondria for autophagic degradation, promoting their clearance via autophagy to maintain mitochondrial quality and function [85]. Mutations or dysregulation of the FUS protein can lead to disruptions in mitochondrial axon transport, and downregulating the expression of Parkin can ameliorate the neurodegenerative phenotypes induced by FUS protein, offering a new perspective for developing therapeutic strategies for *FUS*-induced neurodegeneration [86]. In addition, mitochondrial transport and morphological abnormalities are considered to be common pathological features in SOD1 and TDP43 ALS mouse models [87].

### 5.3. Interaction Between ALS and Axonal Transport Disorder

Axonal transport impairment is considered a crucial pathological mechanism in the etiology of ALS, occurring before the degeneration of motor neurons. Research indicates that mutations in genes associated with axonal transport, such as dynactin subunit 1 (*DCTN1*) [88], profilin 1 (*PFN1*) [89], alsin 2 (*ALS2*) [30], *KIF5A* [90,91], and tubulin alpha 4a (*TUBA4A*) [92], can cause impairments in axonal transport, potentially leading to the progression of ALS. For instance, mutations in *KIF5A* can lead to a loss of its autoinhibition, resulting in a constitutively active state, which manifests as a persistently active motor protein, thereby disrupting intracellular trafficking and the viability of neurons [91]. 

**Figure 3:**
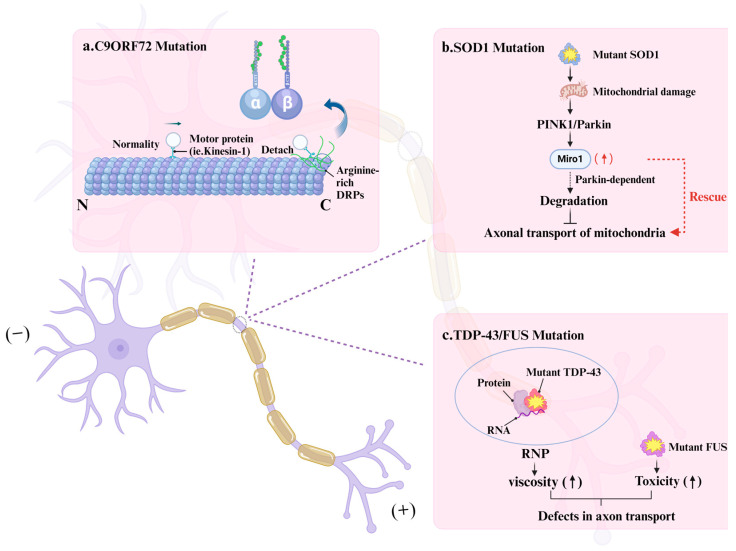
The connection between mutations in the genes that cause ALS and axonal transport disorder. (**a**) The DPRs generated by the *C9ORF72* gene impair the interaction and motility of motor proteins by competitively occupying the binding sites on the C-terminal tail of microtubules. As a result, the presence of DPRs leads to a higher rate of motor protein dissociation from microtubules. (**b**) ALS-associated mutant *SOD1* has been found to inhibit mitochondrial axonal transport by inducing the degradation of Miro1 in a PINK1/Parkin-dependent manner. (**c**) The augmented viscosity of TDP-43-formed ribonucleoprotein (RNP) particles and the heightened toxic gain-of-function of FUS contribute to transport impediments. The blue and black arrows indicate the direction, the curved blue arrows point to the enlarged structure, the dashed red arrows suggest that increased Miro expression can ameliorate the axonal transport function of the mitochondria, and the arrows in parentheses signify trends.

Furthermore, mutations in commonly implicated ALS pathogenic genes have also been proven to cause axonal transport impairments in reverse. The dipeptide repeat sequences (DPRs), particularly poly-PR and poly-GR, arising from the repeat expansion of the *C9ORF72* gene, can directly bind to the exposed C-terminal tails of tubulins. Motor proteins such as kinesin-1 and dynein rely on specific interactions with tubulins to propel their movement, and DPRs impede the binding and motility of these motor proteins by competitively binding to these tails, leading to a decrease in transport efficiency and the accumulation of transport cargo within the axons, which affects the normal neuronal function. Consequently, in single-molecule imaging studies, motor proteins have been observed to pause more frequently or detach from microtubules in the presence of DPRs [93] (Figure 3a). Subsequent research has revealed that reducing the expression of NIMA-related kinase 6 (NEK6) or inhibiting its activity can mitigate the toxicity induced by DPRs due to *C9ORF72* gene mutations, ameliorate axonal pathologies, and rescue axonal transport deficits by reversing p53-associated DNA damage [94]. This discovery paves the way for the development of novel therapeutic strategies targeting C9ORF72-associated frontotemporal dementia (FTD) and ALS. 

Research indicates that the ALS-associated mutant *SOD1* inhibits mitochondrial axonal transport by inducing the degradation of Miro1 in a PINK1/Parkin-dependent manner [95] (Figure 3b). Furthermore, the binding of Ca^2+^ to Miro1 impedes mitochondrial transport by modulating its interaction with kinesin-1 [95]. The mechanism may account for the rapid decline in mitochondrial axonal transport and the simultaneous increase in Ca^2+^ transients observed when motor neurons in microfluidic devices were exposed to conditioned media from mutSOD1 myotubes [96]. Inhibition of p38 mitogen-activated protein kinase (MAPK) has been shown to ameliorate the defects in retrograde cargo transport within axons in a transgenic mouse model expressing the mutant SOD1^G93A^ [97]. Mutations or abnormal aggregations of TDP-43 [98] and FUS proteins [99] can also affect axonal transport (Figure 3c), and the inhibition of histone deacetylase 6 (HDAC6) has been demonstrated to rescue such deficits [98,99,100]. Below, we will explore each gene in detail and elucidate how mutations linked to ALS (presented in Table 2) may disrupt the normal functionality of the respective proteins, along with the ramifications for the axonal cytoskeletal system.

## 6. Axonal Regeneration as a Potential Therapeutic Target for ALS

Axon regeneration is the process through which the axons of neurons regrow and re-establish their functional connections following nerve damage. Research has demonstrated that in conditions such as ALS and other diseases characterized by axonal degeneration, the development of medications that facilitate axonal regeneration could potentially decelerate the progression of symptoms and extend the preservation of muscle function [101]. However, in contrast to the PNS, neurons within the CNS exhibit a markedly diminished capacity for axonal regeneration post-maturity [102]. Consequently, we will delve into therapeutic interventions that may enhance axonal regeneration within the CNS.

### 6.1. Ameliorating the Inhibitory Microenvironment 

For many years, research into the microenvironment that inhibits axonal regeneration in the CNS has been a central focus. Within the CNS, non-neuronal cell types such as microglia, macrophages, oligodendrocytes, and astrocytes create a microenvironment that is not conducive to effective axonal regeneration through various mechanisms [103]. In addition, myelin debris from axonal degeneration may inhibit axonal growth by releasing inhibitory molecules, depleting or sequestering neurotrophic factors, while the clearance of myelin debris by microglia and CNS-infiltrated macrophages is slow [104]. 

Neurite outgrowth inhibitor A (Nogo-A), an axon growth inhibitor expressed by oligodendrocytes, is a protein integral to the CNS and plays a pivotal role in the processes of neural regeneration and plasticity. In a mouse model of ALS, the genetic deletion of Nogo-A has been shown to extend survival and mitigate muscle denervation [105]. Furthermore, studies utilizing the GAL4-VP16/upstream activating sequences (GAL4-VP16-UAS) transgenic zebrafish model have provided compelling evidence that dysfunction in mature oligodendrocytes, triggered by mutSOD1, can precipitate motor neuron degeneration. These insights broaden the spectrum of potential animal models for research and pave the way for novel therapeutic strategies aimed at targeting oligodendrocytes [106].

Chondroitin sulfate proteoglycans (CSPGs), a major component of the extracellular matrix in the mammalian CNS, accumulate in the microenvironment of degenerating motor neurons and physically inhibit axonal regeneration by forming a glial scar [107,108]. From the pre-symptomatic stages of ALS, there is a reduction in neurons and an abnormal increase in the expression of protein tyrosine phosphatase receptor type (PTPr) and leukocyte antigen related (LAR) in astrocytes. In the nervous system, the binding of PTPr and LAR to CSPGs can activate intracellular signaling pathways, such as the Ras homolog gene family, member A (RhoA)/Rho-associated protein kinase (ROCK) pathway, thereby inhibiting the growth and regeneration of axons [109]. Notably, the GTPase RhoA has a cell type-specific dual role in axonal regeneration after CNS injury; activation of RhoA in neurons prevents axonal regeneration, while RhoA in astrocytes is beneficial to regenerating axons [110]. ROCK inhibitors are a promising class of disease-modifying therapy drugs for ALS [111]. Building on these findings, preclinical research has demonstrated that fasudil not only enhances the survival of motor neurons [112] but also plays a crucial role in preventing axonal degeneration and stimulating axonal regeneration [113]. Following these promising preclinical results, fasudil has now advanced to phase II clinical trials [114], marking a pivotal step towards potential therapeutic applications.

Remarkably, Louit et al. constructed a 3D in vitro model that includes motor neurons, astrocytes, microglia, and other cells [115], providing new insights for testing different cell combinations to explore more effective ALS therapeutic strategies. In addition to the aforementioned non-neuronal microenvironment, niclosamide has been shown to reduce inflammation and fibrosis associated with axonal injury in ALS models by inhibiting the S100A4 protein, thereby improving the inflammatory microenvironment and providing more favorable conditions for axonal repair and regeneration [116].

### 6.2. Improving Growth Cone Formation in Damaged Areas

A pivotal indicator for initiating CNS axonal regeneration is the capacity of growth cones to form in the area of injury. Growth cones are adept at sensing and interpreting complex environmental signals, decoding these inputs through molecular mechanisms to provide precise directional guidance for axonal growth [117].

Notably, the axonal repulsion protein Semaphorin3A, upon binding to its receptor neuropilin 1 (NRP1), can induce the collapse and paralysis of growth cones, thereby impeding the growth and regeneration of spinal motor neurons [118,119]. Conversely, Semaphorin5a and Semaphorin6d, signaling proteins within the same family, are upregulated following spinal cord injury. The genetic deletion of these molecules or their receptors, such as neuropilin1 and plexinA1, can suppress the retraction or degeneration of damaged corticospinal neurons, controlling the levels of signaling molecules around the injured neurons and thus creating an environment that is less conducive to axonal regeneration [120]. These findings underscore the remarkable role of the Semaphorin family in the process of axonal regeneration, suggesting their potential as therapeutic targets for enhancing regenerative processes in ALS.

### 6.3. Activating Autophagy

Dual leucine zipper kinase (DLK-1) is an essential and sufficient factor for the activation of autophagy induced by injury. It promotes axonal regeneration, particularly in the context of aging, by restricting the Notch signaling pathway through autophagy activation [121]. This research underscores the potential of autophagy activation in facilitating axonal regeneration, offering a promising therapeutic strategy for recovery after axonal injury, especially when neurons have diminished regenerative capacity. Moreover, the deletion of DLK exhibits a potent synergistic effect when combined with the expression of activating transcription factor 3 (*ATF3*), a master regulatory factor for axonal regeneration [122]. The combined therapy of DLK gene knockout and *ATF3* overexpression could effectively treat neurodegenerative diseases, proving more efficacious than solely preventing cell death or promoting regeneration. Interestingly, *ATF3*, along with other regeneration-associated transcription factors such as CAMP responsive element modulator (*Crem*), AT-rich interactive domain 5A (*Arid5a*), FOS like 1, AP-1 transcription factor subunit (*Fosl1*), and KLF transcription factor 6 (*Klf6*), forms a network that activates a multitude of regeneration-associated genes, furthering axonal regeneration [123]. 

### 6.4. Other Treatment Targets

Research indicates that the dysfunction of TDP-43 leads to a deficiency in the stathmin2 (STMN2) protein, which may impair the regeneration and repair of motor neuron axons [124]. Significant efforts have been concentrated on reducing TDP-43 levels and restoring STMN2. For instance, the c-Jun N-terminal kinase (JNK) inhibitor SP600125 has been utilized to address the growth defects and STMN2 protein deficiency that arise following TDP-43 depletion [125]. Furthermore, targeting deactivated Cas13d family ribonuclease effector (dCasRx) or employing antisense oligonucleotides (ASOs) has shown the potential to rapidly restore axonal regeneration and STMN2-dependent lysosomal transport in TDP-43-deficient human motor neurons [126]. QRL-201 is a first-in-class molecule capable of mitigating the loss of function of STMN2 in models of motor neuron disease, particularly in the context of TDP-43 pathology. Notably, this ALS therapeutic candidate, QRL-201, has successfully advanced into phase I clinical trials.

Despite numerous previous efforts to enhance axonal regeneration (Table 3), including magnetic stimulation [127], stem cell therapy [128], androgen therapy [129], and pharmacological inhibitions such as β-site amyloid precursor protein cleaving enzyme (BACE) inhibitors [130], there remains a significant journey ahead to achieve CNS axonal regeneration in ALS. 

## 7. Conclusions and Outlook

ALS is the most prevalent form of motor neuron disease. Despite increasing attention in recent years, it still lacks effective treatments. There are many factors that lead to the slow progress of disease research, such as the difficulty of early diagnosis, unclear pathogenesis, and a lack of appropriate animal models and effective treatment. To address these challenges, we have reviewed the fine structure of axons and a series of axonal biological processes, including axonal degeneration, axonal transport, and axonal regeneration, with an emphasis on their close relationship with ALS. In particular, we discussed the mutual inhibitory effects between the mutations of common ALS pathogenic genes (*SOD1*, *C9ORF72*, *FUS*, and *TARDBP*) and axonal dysfunction. Understanding the pathogenesis of ALS and the interconnections among the various factors leading to axonal dysfunction is essential for developing more effective treatments. 

The connection between axons and ALS is a highly dynamic process. Axonal degeneration is a critical early event in the pathogenesis of ALS. Impaired axonal transport prevents essential organelles or proteins, such as the axonal key protective factor NMNAT2, from reaching the axon terminals effectively, disrupting their supply to the distal axon, and their depletion can lead to degeneration. Additionally, axonal degeneration can reduce neurotrophic factors, which are crucial for maintaining neuronal survival and health. Over time, the accumulation of axonal damage leads to further degeneration and death of motor neurons. Therefore, developing effective drugs for ALS that target axons should not focus solely on a single aspect of axonal biology; this may be one reason why many drug trials have not been successful. Technologically, researchers have developed a method using single-photon emission computed tomography (SPECT) to quantify net retrograde axonal transport for functional imaging in living animals [131]. This method has broad potential clinical applications, allowing for the early detection of the benefits of potential treatments. It complements microfluidic devices [132], offering a more comprehensive biomedical research approach for the diagnosis and treatment of ALS. 

In conclusion, a multitude of animal studies have identified axons as a promising therapeutic target for ALS. However, the transition from these findings to clinical application necessitates thorough validation in human subjects to determine their relevance in the development of therapeutic interventions. This validation process is essential for offering ALS patients the prospect of novel therapeutic options.

## Figures and Tables

**Table 2:** Genes implicated in axonal transport dysfunction in ALS.

Gene	Mechanism	Refs.
*DCTN1*	The G59S mutation within the glycine-rich domain of the cytoskeleton-associated protein *DCTN1* diminishes the binding capacity of dynein to microtubules	[88]
*PFN1*	Mutations in *PFN1* lead to reduced levels of actin binding, potentially altering cytoskeletal pathways and disrupting normal axonal transport functions	[89]
*ALS2*	In SOD1 (H46R) mice, the ablation of the *ALS2* gene heightens lysosomal transport disruptions, ultimately intensifying the disease phenotype	[30]
*KIF5A*	The *KIF5A* mutation results in the loss of its autoinhibition, leading to a constitutively active state of the motor protein. This sustained activity can impair intracellular transport mechanisms	[90,91]
*TUB4A*	*TUBA4A* mutants destabilize the microtubule network and reduce its reaggregation capacity, resulting in impaired axon transport by affecting microtubule stability and function	[92]
*C9ORF72*	The DPRs produced by *C9ORF72* inhibit the binding and movement of motor proteins by competitively binding to the C-terminal tail of these tubulin exposed tails, resulting in reduced transport efficiency and accumulation of transport cargo in the axon	[93]
*SOD1*	The mutant *SOD1* disrupts the transport of mitochondria within axons by triggering the degradation of Miro1 through a PINK1/Parkin-dependent mechanism and by binding calcium ions to Miro1, which modulates its interaction with kinesin-1	[95]
*TARDBP*	RNP particles formed by TDP-43 mutation form highly viscous aggregates that exhibit destructive transport dynamics	[98]
*FUS*	Mutations or dysregulation of the FUS protein can lead to disruptions in mitochondrial axonal transport, and downregulating the expression of Parkin can ameliorate the neurodegenerative phenotypes induced by FUS protein	[86,99]

**Table 3:** Therapies based on improving axon regeneration in ALS.

Categories	Therapeutic Targets/Strategies	Main Effects	Refs.
Ameliorating the inhibitory microenvironment	Nogo-A	The genetic deletion of Nogo-A has been shown to extend survival and mitigate muscle denervation	[105]
	ROCK	ROCK inhibitor Fasudil, which can increase the survival rate of motor neurons, inhibit axonal degeneration and enhance axonal regrowth both in vitro and in vivo, showing good effects in initial use	[112,113]
	Niclosamide	Inhibition of the S100A4 protein reduces inflammation and fibrosis associated with axonal injury in ALS, ameliorating the inflammatory microenvironment and thereby providing more favorable conditions for axonal repair and regeneration	[116]
Improving growth cone formation in damaged areas	The Semaphorin family	The Semaphorin family, especially Sema3A in combination with Nrp1, induces collapse and paralysis of growth cones, thereby affecting the growth cones of spinal motor neurons and preventing axon growth and regeneration	[118,119,120]
Activating autophagy	DLK-1	Activating autophagy to restrict the Notch signaling pathway, thereby promoting axonal regeneration	[121]
Other treatment targets	JNK	STMN2 protein and growth defects after TDP-43 consumption were remedied by the JNK inhibitor SP600125	[125]
	Targeting dCasRx/ASOs	Restoring axonal regeneration and STMN2-dependent lysosomal transport in TDP-43-deficient human motor neurons within a short-term period	[126]
	Magnetic stimulation	Restoring mitochondrial and lysosomal axonal transport and axonal regrowth sprouting, showing no apparent harmful effects on diseased and healthy neurons	[127]
	Stem cell therapy	Acting as neuronal intermediaries, they receive connections from the regenerated host axons and extend their own axons to connect with the host, including the motor axons in the ventral roots	[128]
	Androgens	Demonstrating neuroprotective effects on the brain and stimulating axonal regeneration	[129]
	Pharmacological BACE Inhibition	Enhancing axonal regeneration and improving muscle reinnervation	[130]

## Data Availability

Not applicable.

## Abbreviations

ADPR	ADP-ribose
*ALS*	amyotrophic lateral sclerosis
*ALS2*	ALSin 2
AP-1	Activation protein-1
*Arid5a*	AT-rich interactive domain 5A
ARM	Armadillo/HEAT motif
ASOs	antisense oligonucleotides
*ATF3*	activating transcription factor 3
BACE	β-site amyloid precursor protein cleaving enzyme
*C9* *ORF72*	chromosome 9 open reading frame 72
cADPR	cyclic ADP-ribose
CNS	central nervous system
*Crem*	CAMP responsive element modulator
cryo-EM	cryo-electron microscopy
CSF	cerebrospinal fluid
CSPGs	chondroitin sulfate proteoglycans
dCasRx	deactivated Cas13d family ribonuclease effector
*DCTN1*	Dynactin subunit 1
DLK-1	Dual-Leucine zipper Kinase
DPRs	dipeptide repeat sequences
DR	dorsal root
DRG	dorsal root ganglia
DTI	diffusion tensor imaging
dWnk	Drosophila Wnk kinase
*Fosl1*	FOS like 1, AP-1 transcription factor subunit
FTD	frontotemporal dementia
*FUS*	fused in sarcoma
GAL4-VP16-UAS	GAL4-VP16/upstream activating sequences
GWAS	genome-wide association study
HDAC6	histone deacetylase 6
IFs	intermediate filaments
IMM	inner mitochondrial membrane
JNK	c-Jun N-terminal kinase
*KIF5A*	Kinesin family member 5A
*Klf6*	KLF transcription factor 6
LAR	Leukocyte Antigen Related
MAPK	mitogen-activated protein kinase
MFs	microfilaments
Miro	mitochondrial Rho GTPase 1
MTs	microtubules
NAD^+^	nicotinamide adenine dinucleotide
NAM	nicotinamide
NaMN	nicotinic acid mononucleotide
NAMPT	nicotinamide phosphoribosyltransferase
NCT	nuclear-cytoplasmic transport
NEK6	NIMA-related kinase 6
NF-H	the neurofilament heavy proteins
NF-L	the neurofilament light proteins
NF-M	the neurofilament medium proteins
NFs	Neurofilaments
NMN	Nicotinamide mononucleotide
NMNAT	Nicotinamide mononucleotide adenylyltransferase
Nogo-A	neurite outgrowth inhibitor A
OMM	outer mitochondrial membrane
*PFN1*	profilin 1
PNS	peripheral nervous system
PTPr	protein tyrosine phosphatase receptor type
RhoA	Ras homolog gene family, member A
ROCK	Rho-associated protein kinase
SAM	sterile alpha motif
SARM1	sterile alpha and Toll/interleukin-1 receptor motion-containing protein 1
SNPH	syntaphilin
*SOD1*	superoxide dismutase
SPECT	single-photon emission computed tomography
STMN2	Stathmin2
*TARDBP*	TAR DNA-binding protein
TIR	the Toll/interleukin-1 receptor
*TUBA4A*	tubulin alpha 4a
UMNs	upper motor neurons
VR	ventral root
